# Impact of the Use of a Standardized Guidance Tool on the Development of a Teaching Philosophy in a Pharmacy Residency Teaching and Learning Curriculum Program

**DOI:** 10.3390/pharmacy4010009

**Published:** 2016-01-26

**Authors:** Amber R. Wesner, Ryan Jones, Karen Schultz, Mark Johnson

**Affiliations:** 1 Department of Pharmacy Practice, Shenandoah University, 1775 North Sector Court, Winchester, VA 22601, USA; rjones11@su.edu (R.J.); mjohnson@su.edu (M.J.); 2 Center for Public Service and Scholarship, Shenandoah University, 1460 University Drive, Winchester, VA 22601, USA; kschultz@su.edu

**Keywords:** teaching philosophy, residency teaching program, teaching certificate

## Abstract

The purpose of this study was to evaluate the impact of a standardized reflection tool on the development of a teaching philosophy statement in a pharmacy residency teaching and learning curriculum program (RTLCP). Pharmacy residents participating in the RTLCP over a two-year period were surveyed using a pre/post method to assess perceptions of teaching philosophy development before and after using the tool. Responses were assessed using a 5-point Likert scale to indicate level of agreement with each statement. For analysis, responses were divided into high (strongly agree/agree) and low (neutral/disagree/strongly disagree) agreement. The level of agreement increased significantly for all items surveyed (*p* < 0.05), with the exception of one area pertaining to the ability to describe characteristics of outstanding teachers, which was noted to be strong before and after using the tool (*p* = 0.5027). Overall results were positive, with 81% of participants responding that the reflection tool was helpful in developing a teaching philosophy, and 96% responding that the resulting teaching philosophy statement fully reflected their views on teaching and learning. The standardized reflection tool developed at Shenandoah University assisted pharmacy residents enrolled in a teaching and learning curriculum program to draft a comprehensive teaching philosophy statement, and was well received by participants.

## 1. Introduction

A teaching philosophy statement is a constantly evolving, reflective document that allows the current or future pharmacy educator to identify and define their fundamental beliefs about teaching and learning, set student-centered teaching goals, and describe their strategies for personal development [1]. The teaching philosophy statement is usually one to two pages in length, and is considered to be an essential part of the comprehensive teaching portfolio of educators, which also contains examples of teaching effectiveness such as handouts, electronic presentations, and assessment items as well as student and peer evaluations of teaching [1]. The inclusion of a teaching philosophy in the teaching portfolio is supported by the American Association of Colleges of Pharmacy (AACP), which highlighted, in a 2012 report by the Academic Affairs Standing Committee, the necessity for a well-articulated teaching philosophy as a crucial component of faculty evaluation [2]. Although not all pharmacists who complete a postgraduate residency program go on to accept full-time positions in academia, studies have shown that most will be involved in some aspect of teaching during their careers, including precepting pharmacy students and residents, and as such, developing a teaching philosophy is widely applicable to a pharmacist’s career [3,4,5].

In the late 1990s, the University of Kentucky implemented the first pharmacy residency teaching certificate program (TCP) [6]. Following the success of this innovative idea many other schools of pharmacy and residency programs subsequently developed similar programs. However, each teaching certificate program is unique, with no accrediting body that sets specific standards, nor is there consistency between programs with regard to requirements for completion or quality of pedagogical content. Despite these differences, it is common to find the formation and writing of a teaching philosophy to be a similarity between programs [5,7,8]. Several descriptive studies involving participants in these programs have acknowledged the perceived importance of developing a teaching portfolio and philosophy statement. A survey of 48 pharmacy residents enrolled in the Indiana Pharmacy Teaching Certificate Program (IPTeC) revealed that 81% believed the development of a teaching portfolio, including a teaching philosophy, to be of high importance [4]. Another survey of current and former residents who graduated over eleven years from a teaching certificate program through the University of Wisconsin—Madison (*n* = 95) found that a solid majority, 63.2%, felt that the creation of a teaching philosophy was probably or definitely important to teaching development [7]. In 2004, fourteen residents affiliated with the Massachusetts College of Pharmacy were subjected to a seminar series on teaching as part of their residency experience, which included a session on evaluation and development of a teaching philosophy. At the end of the series, overall impressions of the program were positive, and 93% of the participating residents reported improved knowledge in formation of a teaching philosophy [8].

The need for formal, structured training for pharmacy residents on how to teach effectively has been suggested in the literature, and may be vital to meeting the American Society of Health-System Pharmacists (ASHP) accreditation standards. In a 2013 article published in the American Journal of Pharmaceutical Education (AJPE) by Wanat *et al.* [9] the researchers found that many pharmacy residents may not be well prepared to transition into clinical faculty roles. Therefore, they concluded that schools of pharmacy should allocate resources to developing the teaching and scholarship skills of their affiliated residents in order to better prepare these future colleagues for the roles they will be increasingly asked to fill. Although ASHP accreditation standards for PGY1 residency programs require graduates to be equipped to successfully deliver educational programs and instruction, there is currently no standardized curriculum for earning a teaching certificate during pharmacy residency, much less for developing a teaching philosophy, and approaches vary between institutions [10,11,12,13].

One common theme noted to be necessary to improving educational effectiveness is introspection [14,15,16]. Commenting on undergraduate and graduate-level education, Donnelly has noted that frequent self-reflection can aid professional educators in continuously improving their instructional approaches, and that systematic, purposeful review of one’s teaching philosophy, methods, and efficacy ensures optimal student outcomes [14]. Unfortunately, other than encouraging frequent self-reflection there is a paucity of information to be found in the medical literature about how to best develop a teaching philosophy or whether a standardized approach is beneficial to this process, especially within the context of pharmacy education. Validated instruments such as the Teaching Perspectives Inventory (TPI) and the Pharmacist’s Inventory of Learning Styles (PILS) have been used to evaluate and compare the preferred teaching and learning styles of pharmacy preceptors and residents [3]. This research, however, does not provide insight into the teaching philosophies that underlie these teaching and learning styles. In fact, according to the 2014 ACPE and ASHP joint recommendations for postgraduate pharmacy training in teaching and learning, the development of a teaching philosophy represents foundational knowledge that is necessary before the resident may graduate into application and practice-based teaching [12,13]. And though the development of a teaching philosophy appears to be a pre-requisite for becoming an effective educator, no instrument appears to have yet been widely used to build this important foundation.

Due to the conspicuous absence of studies involving standardized tools for development of a teaching philosophy, and the vital importance of this process for pharmacy residents, we sought to examine the impact of a standardized reflection tool on residents’ perspectives and ability to create a teaching philosophy. It is the purpose of this article to describe how a teaching philosophy is developed during the Shenandoah University Pharmacy Residency Teaching and Learning Curriculum Program, and to assess the effectiveness of the standardized reflection tool.

## 2. Experimental Section

### 2.1. Methods

In the spring of 2013, the teaching philosophy reflection tool was developed by faculty of the RTLCP as a result of requests by RTLCP participants for more concrete guidance on how to best approach writing a teaching philosophy statement. RTLCP faculty looked for ways to better guide participants in development of their teaching philosophies. In addition to receiving the tool, participants were included in a one-hour discussion regarding the purpose and components of a teaching philosophy with a faculty member where pertinent readings were discussed. The text used for the teaching program was *Pharmacy Education—What Matters in Learning and Teaching* [17]. Also, previously completed teaching philosophies were not provided to the participants in order to ensure original work. However, participants could seek out completed teaching philosophies during their own research. After two years of providing the tool to participants a retrospective analysis of the teaching philosophy standardized reflection tool was conducted. The teaching philosophy reflection tool is displayed in the Appendix. The reflection tool consists of five written activities, and was based on guidance provided in the AACP White Paper regarding “Guidelines for Residency Teaching Experiences” [13]. The first activity asks the resident to recall an outstanding educator, and organize characteristics or actions that made this educator exceptional into a table. The resident is then asked to identify how these characteristics may assist in learning, and how the resident may apply these characteristics to their own teaching.

Activity two consists of three questions regarding views of adult learners. Residents are asked to identify their beliefs about adult learners with regard to educational readiness, or lack thereof, and what adult learners bring to the table. Residents are also asked to identify pedagogies or strategies they feel best serve adult learners, taking into consideration the core beliefs identified in question one, and finally participants list proposed methods to assess their teaching and learning.

The third activity asks the resident to identify his or her roles as a teacher, including how the resident views his or her place in the process of teaching, as well as how learning is best facilitated. Participants are then asked to place a checkmark next to each role that is considered to be a strength. For any role that is not viewed as a strength, the resident is asked to write what he or she would need to do to make the non-checked items into strengths.

Activity four consists of four questions that are designed to be answered while reflecting on Activities one through three. Participants must express their motivation for teaching, identify any assumptions that were made in the completion of each of the prior activities, postulate on whether any points may change as the resident gains more teaching experience, and also asks the resident their plan to continually growth as a teacher.

The fifth and final activity consists of two parts. Part 1 defines the elements of a teaching philosophy: (1) goals and values as a teacher; (2) a description of approaches and methods; (3) assumptions about teaching and learning; and (4) assessment measures to determine teaching effectiveness. In part 2, the resident is asked to reflect on all completed activities and write an introductory paragraph for their teaching philosophy statement.

### 2.2. Data Collection

Participants were surveyed in a pre/post design to assess effectiveness and satisfaction with the reflection tool during the past two years. All participants were PGY1 residents with the exception of three participants. One was a PGY2 and two were practicing pharmacists. Survey questions required participants to rank themselves on a 5-point Likert scale where one indicated strong disagreement and five indicated the highest level of agreement with the survey statement. Survey questions are presented in Table 1. The survey asked questions specific to the teaching philosophy reflection tool. A different survey regarding the teaching program as a whole was administered to participants separately. Surveys were optional to complete, but highly encouraged. The survey tool was meant to illicit feedback regarding the tool to allow for quality improvement, but failure to complete the survey did not prevent participants from earning their teaching certificate.

**Table 1:** Pre and post survey results.

Question Text	Pre-survey (*n* = 35)		Post-survey (*n* = 26)		*p*-Value
	SD/D/N ^a^ (n)	A/SA ^b^ (n)	SD/D/N ^a^ (n)	A/SA ^b^ (n)	
Q1: I understand the purpose of a teaching philosophy.	16	19	1	25	0.0003
Q2: I can write an effective teaching philosophy statement.	27	8	2	24	0.0003
Q3: I know the main categories or elements that should go into a teaching philosophy statement.	32	3	3	23	<0.0001
Q4: I know the questions I need to ask myself to get started in writing and developing a teaching philosophy statement.	27	8	4	22	<0.0001
Q5: I can identify characteristics of outstanding teachers I have had in the past.	2	33	0	26	0.5027
Q6: I can identify beliefs about adult learners whom I have taught in the past.	18	17	1	25	<0.0001
Q7: I can identify strategies or techniques that I can use to best teach adult learners.	21	14	2	24	<0.0001
Q8: I can clearly describe my role as a teacher.	18	17	1	25	<0.0001
Q9: I can identify assessment methods to know that my teaching is effective.	24	11	1	25	<0.0001
Q10: I can describe the responsibilities I will hold myself accountable for as a teacher.	9	26	0	26	0.0074
Q11: I can describe the responsibilities I will hold my students accountable for.	11	24	0	26	0.0015

Notes: ^a^ SD/D/N = Strongly disagree/disagree/neutral; ^b^ A/SA = Agree/strongly agree.

### 2.3. Data Analysis

For statistical analysis, survey answers were stratified into Group A and Group B. Group A consisted of responses of strongly disagree, disagree and neutral (SD/D/N), corresponding to scores of one through three on the Likert scale. Group B consisted of responses of agree and strongly agree (A/SA), corresponding to scores of four and five on the Likert scale. After categorizing the data in this way, data were analyzed using Fisher’s exact test. A *p*-value < 0.05 was considered significant. Data were analyzed using SPSS (SPSS statistics, version 22). This study was conducted in accordance with the Declaration of Helsinki and was approved by Shenandoah University’s Institutional Review Board (Project code: #50).

## 3. Results and Discussion

### 3.1. Results

A total of thirty-eight residents participated in the RTLCP over the two years surveyed. For academic year 2012–2013, ten participants completed the pre-survey and all ten participants completed the post-survey for a response rate of 100%. For the academic year 2013–2014 twenty-eight participants completed the program, twenty-five of which completed the pre-survey and sixteen completed the post-survey representing a response rate of 89% and 57%, respectively. The average response rate over both years was 92% for the pre-survey and 68% for the post-survey. Table 1 summarizes the change in pre and post survey scores for all participants. For all but one question a statistically significant improvement was noted, showing a shift from disagreeing or being neutral with each statement on the pre-survey to agreeing or strongly agreeing on the post-survey (*p* < 0.05). The only question that did not show improvement after use of the tool was question five which pertained to the ability to describe characteristics of outstanding educators (*p* = 0.5027). Most participants already felt strongly that this was something they were able to do prior to using the tool. However, the median score for this question did increase from a four to a five after use of the tool indicating the ability did increase slightly. For the pooled data set, mean response scores increased for all questions, and median scores increased for all except three questions, which did not change between the pre and post surveys. Means and medians are displayed in Table 2.

**Table 2:** Pre and post survey mean and medians.

Question Text	Pre-survey (*n* = 35)		Post-survey (*n* = 26)	
	Mean ^a^	Median ^a^	Mean ^a^	Median ^a^
Q1: I understand the purpose of a teaching philosophy.	3.3	4	4.3	4
Q2: I can write an effective teaching philosophy statement.	2.5	2	4.1	4
Q3: I know the main categories or elements that should go into a teaching philosophy statement.	2.3	2	4.0	4
Q4: I know the questions I need to ask myself to get started in writing and developing a teaching philosophy statement.	2.4	2	4.3	4
Q5: I can identify characteristics of outstanding teachers I have had in the past.	4.3	4	4.6	5
Q6: I can identify beliefs about adult learners whom I have taught in the past.	3.3	3	4.2	4
Q7: I can identify strategies or techniques that I can use to best teach adult learners.	3.1	3	4.3	4
Q8: I can clearly describe my role as a teacher.	3.3	3	4.2	4
Q9: I can identify assessment methods to know that my teaching is effective.	3.0	3	4.1	4
Q10: I can describe the responsibilities I will hold myself accountable for as a teacher.	3.7	4	4.4	4
Q11: I can describe the responsibilities I will hold my students accountable for.	3.7	4	4.4	4

Notes: ^a^ 1 = strongly disagree; 2 = disagree; 3 = neutral; 4 = agree; 5 = strongly agree.

Post surveys across both years included three additional questions, the responses to which are summarized in Table 3. Overall, 81% of surveyed residents answered that they agreed or strongly agreed that the teaching philosophy tool helped them write an effective teaching philosophy statement. Additionally, 96% of residents surveyed believed that the teaching philosophy statement they created using the tool fully reflected their views of teaching and learning, while 92% indicated that providing examples of teaching philosophies was helpful while drafting their own teaching philosophy.

**Table 3:** Additional questions included only on post survey—% response by category.

Question Text	SD/D/N ^a^ (%)	A/SA ^b^ (%)
Q12: My current teaching philosophy statement fully reflects my personal view of teaching and learning.	4	96
Q13: It was useful to have examples of teaching philosophy statements when writing my teaching philosophy statement.	8	92
Q14: The teaching philosophy tool/worksheet helped me write an effective teaching philosophy statement.	19	81

Notes: ^a^ SD/D/N = Strongly disagree/disagree/neutral; ^b^ A/SA = Agree/strongly agree.

### 3.2. Discussion

This study sought to describe a teaching philosophy tool developed and used by the RTLCP at Shenandoah University. Additionally, the effectiveness of the tool was assessed using a pre-post survey design. The study included thirty-eight pharmacy residents over two years, and overall survey response rate was good at 92% and 68% for the pre and post surveys respectively. Results were largely positive, with increased mean response scores for all questions, and significantly increased agreement between pre and post survey statements across all questions except one, which asked residents to assess their ability to recall outstanding characteristics of past educators. This ability was assessed to be strong even before using the tool, and reflects residents having the ability to recall outstanding teachers in their educational history independent of the reflection tool. Furthermore, the tool did not specifically guide residents through the process of defining outstanding characteristics, but only asked the residents to list these qualities subjectively. The large proportion of participants who agreed that the tool helped them develop a comprehensive teaching philosophy suggests that although the reflection tool may not have enhanced residents’ ability to list outstanding teaching qualities, the tool did help residents integrate these qualities into their teaching philosophy statements. Using reflection on past educators as a first step in teaching philosophy development, as was facilitated in the present study, has been recommended in other studies [1,18].

In addition, median scores increased for all except three questions surveyed. Participants felt they were able to successfully complete these three skills even before using the tool. Therefore, median scores for questions one, ten and eleven remained unchanged between pre and post survey responses, and may be related to question format. Question one asked if residents understood the purpose of a teaching philosophy. However, understanding the purpose of a teaching philosophy does not necessarily imply understanding the process of developing a teaching philosophy. As such, this item may not clearly assess the influence of the reflection tool on teaching philosophy development. Questions ten and eleven ask residents to identify the responsibilities they will hold themselves and their students accountable for. As residents may define “responsibility” as something independent of a teaching philosophy, these questions may not have effectively measured the desired outcome. It is noteworthy that median scores for questions directly related to the process of teaching philosophy development (questions two through four), and which contained the text “teaching philosophy”, increased from 2 to 4 on the pre and post surveys respectively.

In regards to the solely post survey questions, question number thirteen showed that a majority of participants found having examples of teaching philosophies to be helpful. However, the program did not provide completed examples so as to ensure original work, but participants did have the ability to research completed examples during their own preparation. After reviewing the results of this study the authors have since started providing example teaching philosophies to ensure the examples reviewed by participants are of high quality.

Development of a teaching philosophy is a key element in pedagogy and learning training. This foundational statement may be especially important for pharmacy residents who have little to no formal teaching experience, but will be increasingly asked to take on faculty and preceptor positions. A well-developed teaching philosophy is recommended by AACP as a crucial factor in pharmacy faculty evaluation, and current models of teaching training recommended by ASHP consider the teaching philosophy statement to be a prerequisite for advanced teaching practice. The search of relevant literature revealed that although teaching philosophy development is generally included in pharmacy residency teaching certificate programs, there are few studies describing the outcomes of the various methods employed.

Sylvia and colleagues described the approach to teaching philosophy development for pharmacy residents at the Massachusetts College of Pharmacy in 2003 [8]. In their program, residents attended up to eight 2-h seminars, one of which involved teaching philosophy statements and portfolio development. In the teaching philosophy seminar, residents read three papers describing different perspectives on teaching, and responded with a critique stating whether or not they agreed with the perspective and why or why not. This program used the tutorial approach, described by Smallwood, which focuses on equipping participants to formulate and critique arguments in a small-group discussion format [16]. Although most of the participants reported increased knowledge about teaching philosophy development by the end of the program, residents were not required to complete a teaching philosophy statement for successful completion. Also, in a paper by Medina and Draugalis they describe a 9-step approach to writing a teaching philosophy [19]. They describe the order and sections that should be included in a philosophy, but do not provide interactive and reflective activities for writers to complete. Our tool would be a highly pertinent activity to complete prior to following the advice provided in the paper by Medina and Draugalis [19]. In the RTLCP program at Shenandoah University, participants are required to develop a teaching philosophy to successfully complete from the program. In order to accomplish this, residents are provided with the reflection tool as well as one-on-one feedback, and a majority of the residents surveyed in the present study (81%) believed that the reflection tool was helpful to the process. Other programs described in the literature have required the completion of a teaching philosophy statement to earn a teaching certificate, but have not reported in detail how their programs facilitate its development.

Limitations of the present study include being conducted in a single center, surveying a relatively small sample size, and the subjective nature of self-response surveys. Since participants were provided one on one feedback on their philosophies, in addition to being provided the reflection tool, survey responses could have theoretically been affected. However, given that the nature of the comments provided are requests to expand the scope of their philosophy to include learners outside of pharmacy, or related to grammatical changes the effect on the survey responses is thought to be minimal. As noted above, some questions may have suffered from limited internal validity. Additionally, for the second year of the program, the survey response rate was markedly decreased at 57%, so the perceived utility of the tool for non-responders cannot be assessed. In addition, the present study used pooled, unpaired data, so it is not possible to ascertain individual improvement in scores from the study results; only that as a group. In the future, participants can be asked to enter a study number so pre and post responses can be matched and additional statistical tests could be run. Finally, the present study is unable to draw any conclusions about how the reflection tool impacted quality of teaching, teaching positions, or promotions obtained after completing the RTLCP.

Future studies could seek to discover which components of a reflection tool, such as the one used in this study, are most helpful. Also, additional characteristics of the user should be gathered to assess baseline ability to construct a teaching philosophy, such as level of previous teaching experience, pre-professional undergraduate or graduate degree earned, age, and work experience. Since the present study assessed experience with a single tool, other studies could seek to develop modified tools or methods for comparison. Ratnapradipa and Abrams have suggested viewing the teaching philosophy statement as a fluid document, able to be re-shaped over time by the teaching experiences. In addition, Wahl *et al.* found that the perceived importance of teaching philosophy development and reflection on teaching effectiveness is maintained after completion of a RTLCP and entrance into the work force [7]. Therefore, additional studies should examine the evolution of the teaching philosophy over time, surveying educators early in their career and re-visiting their perspectives after gaining experience.

## 4. Conclusions

A standardized reflection tool, designed to encourage personal and professional reflection on past educational experiences, assumptions about adult learners, and teaching and learning goals assisted pharmacy residents enrolled in a teaching and learning curriculum program to draft a comprehensive teaching philosophy statement, and was well received by participants. The current study establishes the potential utility of standardized guidance tools within pharmacy teaching certificate programs to ensure consistent quality of instruction and learning outcomes for pharmacy residents enrolled in such programs.

## Appendix

Teaching philosophy reflection tool for Shenandoah University residency teaching and learning curriculum program (RTLCP).

### Activity 1: Exploring What YOU Value in Outstanding Teaching

Think of a professor, teacher, or educator who influenced you the most. What was it that he or she did and what are the characteristics that person had to make you feel this way?

List them in the table below:

What other actions or characteristics did some outstanding teachers have that you found helpful to your learning and to your accomplishments?

### Characteristics or Actions of your Outstanding Teacher/Educator

**Characteristic or Action**	**How might this Assist in Learning?**	**How might This Be a Part of Your Teaching?**
**1.**		
**2.**		
**3.**		
**4.**		

### Activity 2: What do you believe about your learners and who are they? What do they bring to the learning environment?

You will have the opportunity to be in many teaching and learning environments. This includes, “…continuing education or other invited presentations, providing education to colleagues regarding clinical initiatives, precepting pharmacy students (introductory and advanced experiences) and residents, and educating other health care professionals.” [20].

What do you believe about *adult* learners? What is it that they bring to the table in terms of readiness, or lack thereof, to learn?

Please list beliefs about who your learners are AND what they bring to the table:
(1)
(2)
(3)
(4)
(5)
Having listed the above, what *techniques, pedagogies or strategies* will you use that would best serve those individuals in their learning?
(1)
(2)
(3)
(4)

How do I know that my teaching methods are effective? How will learning be assessed?
(1)
(2)
(3)
(4)

### Activity 3: Defining Your Role in Teaching:

Now that you have explored who your learners are, what they bring to the table (or what they don’t bring!), and what methodologies or pedagogies you will use, the question is:

How do you view yourself and your place in assisting others to learn? How best can you facilitate that learning?

List what you see your role as a teacher is:
(1)
(2)
(3)
(4)
(5)

Of the above listings, place check marks by what you view as your main strengths.

Of those that might not have check marks, write what you would need to do to make them into strengths.

### Activity 4: Exploring Your Decision of Teaching:

You have enormous options as a pharmacist. Why choose to teach? Looking back at what you have written in this entire document, explore the following and briefly write your responses:
(1) Why do I choose to teach?
(2) What did I assume in coming to these thoughts and conclusions?
(3) Do I anticipate any of my points changing as I gain more experience in teaching?
(4) What will I do to continue growing as a teacher?

### Activity 5: The Final Product—at Least for Now!

Your teaching philosophy statement is *yours* but it can be shared with your students, with those to whom you report, and can be part of your CV. Most importantly, it is documentation of your core beliefs and how you conduct yourself in the honorable profession of teaching. We all continue to grow so your statement should change as you do.

The teaching philosophy format should typically be one to two pages in length. It is a narrative reflection that is written in the first-person voice [20]. It generally should include four primary areas [20]: (1) Your purpose for teaching; (2) A description of your teaching style; (3) Statement regarding your beliefs about learning; (4) A description of how your teaching effectiveness will be measured and how you plan to reflect on your teaching

Review what you have written from this Teaching Philosophy guide. Write a clear, succinct opening introductory paragraph that includes what you will state in your philosophy statement. Ensure that each paragraph has a lead sentence. Most importantly, write the statement that makes sense to you and represents your beliefs in the clearest way possible.

### Optional:

As the educational field develops with greater technology, be open to producing a teaching philosophy statement video. Post it! Certainly art, music, and inclusion of personal note from colleagues and students can become part of your teaching philosophy statement.

Develop an elevator statement which is a verbal statement, less than 1 minute in length that you could entitle, “This I believe”. Embrace your teaching and know you are influencing countless lives in positive ways.
